# Editorial: Cardiac outflow tract development and diseases

**DOI:** 10.3389/fped.2023.1323167

**Published:** 2023-11-09

**Authors:** Marta Camprubí-Camprubí, Joan Sanchez-de-Toledo

**Affiliations:** ^1^ Department of Neonatology, Hospital Sant Joan de Déu, Barcelona, Spain; ^2^ Cardiovascular Research Group, Sant Joan de Déu Research Institute, Barcelona, Spain; ^3^ BCNatal-Barcelona Center for Maternal Fetal and Neonatal Medicine, Hospital Sant Joan de Déu-Hospital Clinic, University of Barcelona, Barcelona, Spain; ^4^ Department of Pediatric Cardiology, Hospital Sant Joan de Déu, Barcelona, Spain; ^5^ Department of Critical Care Medicine, University of Pittsburgh, Pittsburgh, PA, United States

**Keywords:** conotruncal abnormalities, fallot tetralogy, DORV, transposition of great arteries, sub aortic stenosis

**Editorial on the Research Topic** Cardiac outflow tract development and diseases

Conotruncal anomalies are the group of congenital heart defects involving malformations of the outflow tract (conus arteriosus) and the great vessels (truncus arteriosus) that have abnormal ventriculo-arterial relationships. Conotruncal (or outflow tract) defects are among the most common congenital heart diseases found at birth, and are the leading cause of mortality and morbidity in the first year of life. Tetralogy of Fallot (ToF), transposition of the great arteries (TGA), double outlet right ventricle (DORV), truncus arteriosus (TA), interrupted aortic arch (IAA) and anatomically corrected transpositions are some of the most common conotruncal anomalies.

A detailed understanding of the embryogenesis is key to understand the conotruncal anomalies. The role of neural crest and second heart field derivatives has been established during outflow tract development. Thus, defective neural crest or second heart field deployment results in a spectrum of conotruncal anomalies ranging from outflow tract alignment to septation defects (1).

This research topic focuses on recent advances in understanding the pathogenesis, diagnosis, treatment, and outcome of the most common conotruncal malformations. This collection includes four original articles that provide a global and detailed overview of some of the key concepts and recent advances related to these entities. The authors of the collection provide an in-depth review of some of the most important malformations included in this group of defects.

DORV was first described in the mid-sixties. This term does not encompass a single congenital heart defect but rather a wide range of phenotypes (2, 3). Due to its heterogeneity, surgical approaches ranging from univentricular palliative procedures to different biventricular surgical repairs have been proposed. Bell-cheddar et al. have prepared an interesting review of DORV providing a general overview including history, description of key morphologic features and anatomical classifications and, a thorough description of the different surgical options. In their review, the authors highlight the importance of a meticulous morphologic assessment prior to deciding the best-suited surgical technique. Particularly important are those anatomical features related to ventricular alignment, ventricular size and coronary anatomy. A detailed list of the most commonly expected surgical complications as well as a practical approach on pre- and post-surgical management is also provided. Overall, Bell-cheddar et al. have done a beautiful job addressing a complex group of CHD in a very organized and practical approach. Moreover, the excellent iconography supporting this manuscript will help readers to engage and better understand the anatomical classifications and different surgical options.

ToF is the most common cyanotic congenital heart disease (3). As with many other congenital malformations, identification of genetic causes of ToF is key to advance in the counselling of families as well as in the medical care of these patients. Only 20% of cases of ToF are syndromic with an identified pathogenic gene. This group includes DiGeorge syndrome, trisomy 21, Alagille syndrome, Ritscher-Schinzel-like syndrome, and CHARGE syndrome (4). This means that in 80% of cases we do not know whether there is a possible genetic cause (5). In one of the papers in this collection, Harvey et al. extend this knowledge by investigating possible mutations in genes related to myocyte contraction and ventricular septum development in non-syndromic ToF patients. Using one of the largest series of patients who have undergone exome analysis to study *de novo* variants, their analysis revealed a significant enrichment of variants in pathways involved in aortic and ventricular development, including a specific enrichment in patients with ToF. They also show the importance of chromatin remodeling genes in cardiac development and report overlap with genes involved in autism spectrum disorders and neurodevelopmental disorders.

One of the emerging fields in pediatric cardiology is prenatal diagnosis. The identification of cardiac malformations during fetal life has led to an improved survival and perinatal management, as well as increased knowledge and acceptance of the condition by families.

Likewise, fetal cardiology has not only been able to detect and identify almost all congenital heart defects, but has also begun to generate evidence on the prognosis and subsequent outcome of these patients. In this line, Gomez et al. addresses the role of some cord blood biomarkers in two of the main conotruncal entities, D-TGA and ToF, studying their correlation with fetal echocardiography and perinatal outcome. The authors reported increased levels of transforming growth factor β1 (TGFβ, in fetuses with ToF when compared with D-TGA and control fetuses. This cytokine is produced by various cells and plays an essential role in the development of cardiac remodeling and fibrosis. They found a strong correlation between cord blood TGFβ levels and prenatal right ventricular OFT obstruction, especially in ToF patients, suggesting that it could be a great new prognostic tool.

Sub-valvular aortic stenosis (SAS) is a common form of left ventricular outflow tract obstruction that can lead to aortic valve damage. It has a broad spectrum of disease, ranging from a small fibrous ridge on the sub-valvular ventricular septum to a narrow fibromuscular tunnel (6). The main important complication of SAS is aortic regurgitation (AR), which is usually progressive. Although surgery is the most widely accepted treatment, the timing and type of surgical intervention remains controversial because progression of AR after subaortic resection is common. Schelein et al. have published a retrospective review of 103 patients aged less than 18 years, who underwent surgical repair of SAS over the past 35 years.

In their cohort, survival rates were 90.8% at 10 years and 88.7% at 20 and 30 years. Main risk factors for mortality were age less than 1 year at the time of surgery, pre-existing Shone's complex condition, mitral stenosis and concomitant surgery on the mitral valve.

The incidence of reoperation for SAS was 21.6% at 10 years and 28.2% at later stages.

No difference in reoperation was detected regarding the surgical approach. The authors conclude that while most patients have a favorable long-term outcome, the recurrence rate of SAS after surgical repair is not negligible.

In conclusion, the manuscripts included in this collection will certainly help to better understanding the conotruncal anomalies, a complex group of CHD with a wide spectrum of morphological types as well as clinical presentations. Early identifications of critical morphological features and the use of new prognostic tools might play an important role in overall postnatal management. Moreover, expanding our current knowledge on genetics and whole exome sequence might provide new insights in the diagnosis and parental counseling. Finally, innovation and development of new surgical approaches and minimally invasive techniques tailored to specific diseases will help to path the way of this complex group of heart anomalies.
